# Dynamics of Dental Enamel Surface Remineralization under the Action of Toothpastes with Substituted Hydroxyapatite and Birch Extract

**DOI:** 10.3390/ma17092038

**Published:** 2024-04-26

**Authors:** Cristina Teodora Dobrota, Alexandra-Diana Florea, Csaba-Pal Racz, Gheorghe Tomoaia, Olga Soritau, Alexandra Avram, Horea-Rares-Ciprian Benea, Cristina Lavinia Rosoiu, Aurora Mocanu, Sorin Riga, Attila-Zsolt Kun, Maria Tomoaia-Cotisel

**Affiliations:** 1 Research Center of Excellence in Physical Chemistry, Faculty of Chemistry and Chemical Engineering, Babeş-Bolyai University, 11 Arany Janos St., 400028 Cluj-Napoca, Romania; cristina.dobrota@ubbcluj.ro (C.T.D.); diana_florea03@yahoo.com (A.-D.F.); csaba.racz@ubbcluj.ro (C.-P.R.); alexandra.avram@ubbcluj.ro (A.A.); aurora.mocanu@ubbcluj.ro (A.M.); d_s_riga@yahoo.com (S.R.); attila.kun@ubbcluj.ro (A.-Z.K.); 2 Department of Molecular Biology and Biotechnology, Faculty of Biology and Geology, Babeş-Bolyai University, 44 Republicii St., 400015 Cluj-Napoca, Romania; cristina.rosoiu@stud.ubbcluj.ro; 3 Department of Orthopedics and Traumatology, Iuliu Hatieganu University of Medicine and Pharmacy, 47 General Traian Mosoiu St., 400132 Cluj-Napoca, Romania; tomoaia2000@yahoo.com (G.T.);; 4 Academy of Romanian Scientists, 3 Ilfov St., 050044 Bucharest, Romania; 5 Oncology Institute of Cluj-Napoca, 34-36 Republicii St., 400015 Cluj-Napoca, Romania; olgasoritau@yahoo.com

**Keywords:** AFM, calcined substituted hydroxyapatites, enamel, remineralization, toothpastes, roughness and statistical analysis, XRD

## Abstract

To address tooth enamel demineralization resulting from factors such as acid erosion, abrasion, and chronic illness treatments, it is important to develop effective daily dental care products promoting enamel preservation and surface remineralization. This study focused on formulating four toothpastes, each containing calcined synthetic hydroxyapatite (HAP) in distinct compositions, each at 4%, along with 1.3% birch extract. Substitution elements were introduced within the HAP structure to enhance enamel remineralization. The efficacy of each toothpaste formulation was evaluated for repairing enamel and for establishing the dynamic of the remineralization. This was performed by using an in vitro assessment of artificially demineralized enamel slices. The structural HAP features explored by XRD and enamel surface quality by AFM revealed notable restorative properties of these toothpastes. Topographic images and the self-assembly of HAP nanoparticles into thin films on enamel surfaces showcased the formulations’ effectiveness. Surface roughness was evaluated through statistical analysis using one-way ANOVA followed by post-test Bonferroni’s multiple comparison test with a *p* value < 0.05 significance setting. Remarkably, enamel nanostructure normalization was observed within a short 10-day period of toothpaste treatment. Optimal remineralization for all toothpastes was reached after about 30 days of treatment. These toothpastes containing birch extract also have a dual function of mineralizing enamel while simultaneously promoting enamel health and restoration.

## 1. Introduction

Tooth enamel represents the most highly mineralized and robust tissue within the human organism [1]. Its composition consists predominantly of hydroxyapatite (HAP) crystals, constituting 95 wt.%, enveloped by organic matter comprising 1–2 wt.% [2,3]. Notably, the HAP crystals present in enamel surpass the length of those observed in normal bone tissue [4].

Prolonged and regular usage over the course of decades may induce a reduction in apatite crystal length, leading to HAP crystals fracturing into smaller entities [5,6].

Adult enamel lacks cells and, as a result, is incapable of self-regeneration. Moreover, enamel is vulnerable to erosion caused by various processes, including tooth abrasion and acid erosion [7,8,9]. 

Numerous studies discuss the demineralization-related changes within enamel when subjected to acid-etching treatments. Data from the literature shows that acid erosion is the most destructive when being facilitated by citric acid [10], phosphoric acid [11,12,13,14], and acetic acid [15,16]. 

Multiple remineralizing agents have been studied, with fluoride being the preferred one, as it prevents natural demineralization through the formation of fluorapatite, which is less soluble than normal HAP. However, some concerns regarding overexposure to fluoride and fluorosis have been raised [17,18,19]. In recent years, synthetic hydroxyapatite, and more specifically nanohydroxyapatite (nanoHAP), has been proposed as a better alternative due to its high biocompatibility, bioactivity, and similarity to the one already present in enamel. Thus, the effectiveness of HAP regarding remineralization has led to multiple studies discussing its presence in different oral-care products [20,21,22,23,24,25,26,27,28,29,30,31,32]. 

Several toothpaste formulations containing hydroxyapatite are proposed within this paper. The hydroxyapatites in these formulations are substituted with different beneficial elements, such as Zn, Si, Mg, and Sr. For example, zinc blocks the glycolytic enzymes, slowing down the metabolism of the bacteria and reducing their ability to develop [33], which is particularly important for good oral hygiene. In addition, zinc prevents calculus formation [34]. The substitution with magnesium is relevant as this element is required for adequate incorporation of the calcium within teeth. A lack of magnesium would lead to weak enamel regardless of the amount of calcium [35]. Moreover, Mg can also promote dental health through a reduction in oral inflammation and its antimicrobial nature [36]. Silicon was reported to have a role in the remineralization process of demineralized dentin and enamel following in vitro testing [37,38,39]. Lastly, the presence of strontium is reported to decrease the demineralization of enamel while also impeding the loss of surface hardness in more acidic environments [40,41]. 

Many such studies rely on scanning electron microscopy imaging (SEM) [42,43,44,45,46]. atomic force microscopy (AFM) is also a suitable technique for evidencing changes in enamel topography because it can provide more quantitative results on surface roughness. Several articles have been published that use the AFM technology to track erosion and demineralization changes in enamel [47,48,49,50,51,52,53,54,55]. 

This article studies the effect of different toothpaste formulations containing calcined hydroxyapatites (e.g., pure or with lattice substitutions of Ca^2+^ ions with Sr^2+^, Zn^2+^, and Mg^2+^, and SiO_4_^4−^ instead of PO_4_^3−^) on the remineralization of artificially demineralized acid-etched enamel. 

The null hypothesis is that there is no effect resulting from the treatment of acid-etched enamel surfaces by using the following toothpaste formulations: P1, containing HAP-Zn; P2, pure HAP; P3, HAP-Mg-Zn-Sr-Si; and P4, HAP-Mg-Zn- Si.

The main purpose of this study is to determine whether the addition of these ions within the nanoHAP structure would affect the dynamics of the remineralization process when compared to pure stoichiometric hydroxyapatite. Similar research has used commercial toothpastes containing Zn-HAP [56,57], Zn carbonate hydroxyapatite [58,59], nanocarbonate-substituted HAP [60], Zn-Mg-hydroxyapatite [61], and Mg-Sr-carbonate hydroxyapatite [59,62]. 

The novelty of this study consists in the use of nanopowders of calcined multisubstituted HAPs (msHAPs) with Mg, Zn, Sr, and Si synthesized by a wet chemical precipitation approach previously described by us [63,64] to develop four new toothpastes (P1–P4). Along with these HAPs (4%), birch extract (1.3%) was utilized in the toothpaste formulation. Birch extract has garnered attention for its composition rich in components possessing antimicrobial, anti-inflammatory, antioxidant, and antiviral properties. In the context of this experimental study, the birch extract was incorporated into the formulation of toothpaste for its antimicrobial efficacy [65]. The toothpastes were examined in vitro for their potential to remineralize enamel and for antimicrobial properties [66]. The novelty of this work is proven by developing new toothpaste formulations containing calcined hydroxyapatite with these precise combinations and birch extract. Furthermore, to the best of the authors’ knowledge, no studies or commercial products employing toothpaste containing calcined HAP with four elemental substitutions exist, and hence this is one of innovations within this investigation. 

## 2. Materials and Methods

### 2.1. Materials

The following reagents were used: calcium nitrate (Ca(NO_3_)_2_·4H_2_O, >99% purity, Sigma Aldrich, Burlington, MA, USA); diammonium hydrogen phosphate ((NH_4_)_2_HPO_4_, 99.4% purity, Chempur, Piekary Śląskie, Poland); magnesium nitrate hexahydrate (Mg(NO_3_)_2_·6H_2_O, 99% purity, Sigma Aldrich); zinc nitrate hexahydrate (Zn(NO_3_)_2_·6H_2_O), >98% purity, Sigma Aldrich); strontium nitrate (Sr(NO_3_)_2_, 99.995% purity, Sigma Aldrich, Burlington, MA, USA); tetraethyl orthosilicate (TEOS, Si(OC_2_H_5_)_4_, 98% purity, Sigma Aldrich); silicon dioxide (SiO_2_, 99.5% trace metals basis, Sigma Aldrich); sorbitol (C_6_H_14_O_6_, ≥98% purity, Sigma Aldrich); PEG400 (Merck, Darmstadt, Germany); xanthan gum (C_35_H_49_O_29_, Sigma Aldrich); sodium lauryl sulfate (CH_3_(CH_2_)_11_OSO_3_Na, ≥99.0% purity, Sigma Aldrich). All reagents were of analytical grade and were used without any further purification.

### 2.2. Hydroxyapatite Synthesis

For the preparation of the 4 remineralizing bioactive toothpastes, 4 types of different hydroxyapatites (simple, substituted, and multisubstituted HAPs)—HAP [theoretical formula: Ca_10_(PO_4_)_6_(OH)_2_], HAP-5%Zn [theoretical formula; Ca_9.22_Zn_0.78_(PO_4_)_6_(OH)_2_], HAP-0.23%Mg-3.09%Zn-2%Si-10%Sr [theoretical formula: Ca_8.19_Mg_0.10_Zn_0.5_Sr_1.21_(PO_4_)_5.25_(SiO_4_)_0.75_(OH)_1.25_], HAP-2.5%Mg-2.9%Si-1.34%Zn [theoretical formula: Ca_8.80_Mg_1.00_Zn_0.20(_PO_4_)_5.00_(SiO_4_)_1.00_(OH)_1.00_]—were synthesized following a method developed by us [40,63,64,66]. Briefly, two aqueous solutions were used. Solution 1 contained the cations Ca^2+^, Mg^2+^, Zn^2+^, and Sr^2+^ and solution 2 contained the anions PO_4_^3−^ and SiO_4_^4−^ corresponding to the desired HAP. Both solutions were mixed using a peristaltic pump and a type Y reactor to ensure equal flows. The pH value was adjusted to 11.5 by adding ammonia. The mole ratio (Ca, Mg, Zn, Sr)/(P, Si) was maintained at the theoretical value of 1.67, specific to the hydroxyapatite stoichiometry. The obtained suspension was filtered, and the precipitate was washed repeatedly, until nitrate free, with double-distilled water. Finally, the dried precipitate was freeze-dried, thus obtaining the desired hydroxyapatite powders. All samples obtained after freeze-drying were further calcined in an electrical furnace at 300 °C for 1 h and investigated by X-ray diffraction, XRD.

### 2.3. Toothpaste Synthesis 

The following reagents were used to create the four experimental toothpastes: double-distilled water (67 wt.%), silicon dioxide (9 wt.%), sorbitol (7.35 wt.%), PEG400 (7.35 wt.%), hydroxyapatite (4 wt.%), birch extract (1.3 wt.%), xanthan gum (0.4 wt.%), sodium lauryl sulphate (0.2 wt.%), ethyl alcohol (3.7 wt.%). The composition was kept constant for all 4 toothpastes. The only difference was the active compound, hydroxyapatite, namely HAP-5%Zn (P1), HAP (P2), HAP-0.23%Mg-3.09%Zn-2%Si-10%Sr (P3), HAP-2.5%Mg-2.9%Si-1.34%Zn (P4). The toothpastes were produced as follows: (i) Two aqueous dispersions were prepared, one of SiO_2_ and another of hydroxyapatite. The two dispersions were mixed under vigorous stirring on a SHP Steriltechnik LABOMAG Multipoint magnetic stirrer. (ii) Sorbitol was dissolved in double-distilled water. (iii) PEG400 and xanthan gum were added to the sorbitol solution until a paste is obtained. (iv) The paste was added to the HAP and SiO_2_ dispersion under vigorous stirring. (v) Sodium lauryl sulfate was then added with birch extract and the final mixture was vigorously stirred until homogenization was achieved. The obtained toothpaste compositions were deposited in hermetically closed polyethylene flasks and kept at room temperature. 

### 2.4. Enamel Sample Preparation and Treatment Protocol

The study used 21 healthy third molars belonging to adults between 20 and 50 years old, extracted for orthodontic purposes (n = 21). All molars met selection criteria, showing healthy enamel with no cavities or anomalies. After ultrasonic cleansing and sterilization, enamel quality was assessed with a stereomicroscope (Carl Zeiss Stereo 475002, Jena, Germany). The molars were embedded in acrylate prisms (Duracryl Plus, Spofadental Inc., Jicin, Czech Republic) up to the cemento-enamel junction for easier handling, and enamel slices were cut for analysis. 

From each of the 21 molars were obtained 4 longitudinal slices with thickness of 1.5 mm and dimensions of 6 mm × 8 mm, for a total of 84 slices. The samples were randomly divided into different groups. Experiments were carried out in triplicate. 

Natural enamel slices served as the control group (n = 6). The rest of 78 slices were demineralized for 60 s with 37.5% orthophosphoric acid (Gel Etchant, Kerr Dental, Brea, CA, USA) using an acidic etching model. Then, these were divided into a group with artificially demineralized enamel (ADE) without treatment (n = 6) and the test groups (72 slices), with the latter subjected to different P1-P4 toothpaste treatments (n = 6, for each toothpaste), for 10 days (24 slices), 20 days (24 slices), and 30 days (24 slices). Each treatment involved brushing the enamel slices twice daily for 3 min. Samples were collected at the end of each treatment phase for the AFM examination. All samples underwent thorough cleaning before AFM imaging and surface roughness analysis at the Scientific Research Center of Excellence in Physical Chemistry, part of STAR Institute, in Babes-Bolyai University of Cluj-Napoca. 

### 2.5. Analysis Methods

#### 2.5.1. Viscosity Measurements

The viscosity of toothpastes (P1–P4) and two commercial toothpastes (C1–C2) was measured with a Brookfield DV-II+Pro viscometer using #4, and #7 spindles [67,68]. All toothpastes were kept in the same condition. Measurements were performed at a constant temperature of 25 °C and at two spindle velocities, namely 10 and 20 RPM. 

#### 2.5.2. XRD 

Using a D8 advanced diffractometer in Bragg–Brentano geometry with an X-ray Cu tube powered at 40 kV/40 mA, the X-ray powder diffraction patterns were obtained. The Ge (1 1 1) monochromator is mounted in the incident beam in order to obtain only CuKα1 radiation, with λ = 1.54056 Å, and the diffracted radiation was recorded with a LYNXEYE detector. The scan was performed in the 10–80° angular 2θ range, with a scan rate of 0.02°/s [69,70]. 

#### 2.5.3. AFM Investigation 

Atomic force microscopy was employed to monitor the remineralization process as a result of treatment, a complex investigation that combines morphology and enamel surface quality characterized by measuring surface roughness, R_RMS_. The AFM imaging was carried out with a JEOL JSPM 4210 Scanning Probe Microscope manufactured by the Jeol Company, Tokyo, Japan [71,72]. It was operated in tapping mode with MikroMasch Co. (Sofia, Bulgaria) NSC 15 hard cantilevers and a speed rate ranging from 0.5 to 1.5 Hz depending on the scanned area. The cantilever parameters are 325 kHz resonant frequency and 40 N/m force constant. The enamel nanostructure was scanned at 5 µm × 5 µm and its ultrastructure at 1 µm × 1 µm. For each, at least five different areas were observed. 

The obtained images were processed in accordance with standard methods using the specialized software WinSPM Processing 2.0, developed by Jeol Company, Tokyo, Japan [40,63,64]. Topographic images revealed surface morphology, while tridimensional profiles displayed the surface roughness obtained by root mean square, Rrms [73,74]. At least three AFM images obtained for each scanned area of each surface enamel slice were analyzed for morphology and surface roughness. Histograms were processed using Microcal Origin 6.0 (Microcal Software Inc., Northampton, MA, USA).

#### 2.5.4. Statistical Analysis

GraphPad Prism 6 (GraphPad Software, Inc., La Jolla, CA, USA) for Windows was used for statistical analysis. All data of surface roughness, R_RMS_, are defined as the mean value ± SD for at least three independent experiments. Significant differences among groups of enamel slices were identified applying one-way ANOVA analysis followed by post-test Bonferroni’s multiple comparison test with a *p* value < 0.05 significance setting. 

## 3. Results

### 3.1. X-ray Powder Diffraction, XRD 

**Figure 1:**
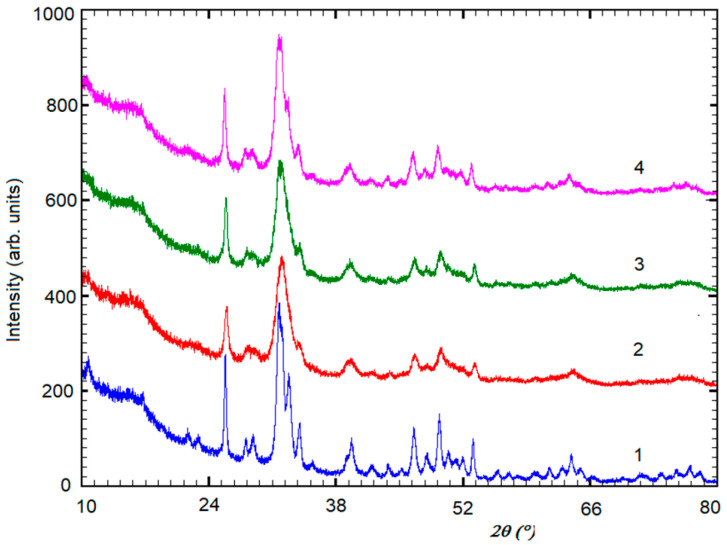
X-ray powder diffraction patterns, XRD, for calcined hydroxyapatites, HAPs, at 300 °C for 1 h: (1) HAP; (2) HAP-Zn; (3) HAP-Mg-Zn-Si; and (4) HAP-Mg-Zn-Sr-Si.

The XRD patterns for the calcined HAPs are shown in Figure 1, for 2θ between 10° and 80°. Phases were identified by comparing the peak positions of the experimental XRD patterns with those of the ICDD (International Centre for Diffraction Data) powder diffraction files (PDF), such as PDF 74-0566 of HAP for stoichiometric hydroxyapatite, HAP-Zn, and HAP-Mg-Zn-Si, and PDF 34-0484 of (Ca)_9_Sr(PO_4_)_6_(OH)_2_ for calcined HAP-Mg-Zn-Sr-Si. Thus, it is proved that these biomimetic HAPs have a high purity for a single HAP structure.

XRD data were used to calculate crystallite sizes, crystallinity degree, and lattice parameters (a = b and c values), which are given in Table 1, for each of investigated hydroxyapatites.

The calculated lattice parameters revealed only slight changes with the compositional modification within the HAP structure (Table 1). The small composition differences lead to a slight distortion in the HAP lattice and, thus, a small drop in its crystallinity is observed by increasing the substitution within the HAP structure. 

### 3.2. Particle Size Distribution and Surface Roughness of the Four HAPs Nanopowders

All four nanohydroxyapatite powders serve as the primary active ingredient in their respective toothpaste formulations. In this context, the particle size distribution is a critical factor affecting remineralization efficacy. Smaller particles possess a larger specific surface area, enhancing their ability to release ions compared to larger particles. However, excessively small nanoparticles may agglomerate, hindering the intended remineralization process. Therefore, prior to incorporating them into toothpaste formulations, the four synthesized nanoHAP samples underwent characterization for size distribution using atomic force microscopy. To accomplish this, all powders were deposited onto glass slides through vertical adsorption from their aqueous dispersions.

**Figure 2:**
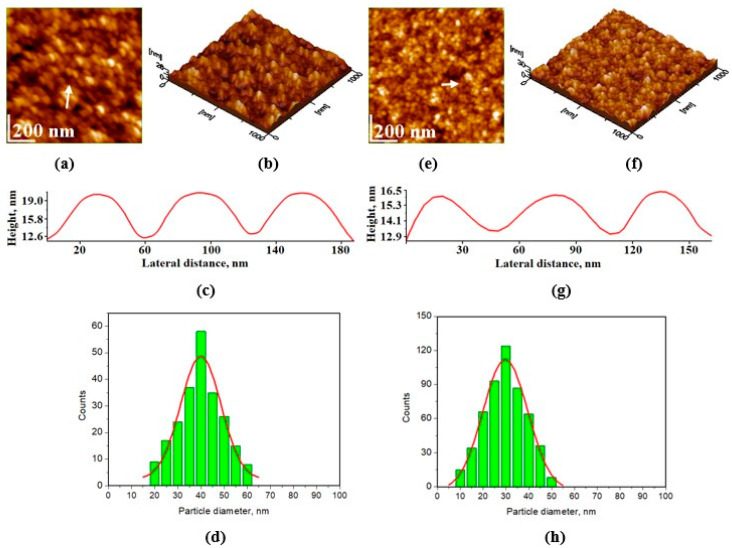
AFM images for HAP-5%Zn used in toothpaste P1: (**a**) 2D- topography image and (**b**) 3D-(tridimensional) image; surface roughness, Rrms = 4.0 ± 1.3 nm, on 1 µm × 1 µm scanned area; (**c**) profile along the arrow in panel (**a**); (**d**) histogram of particle size distribution on image (**a**); the same characteristics: (**e**–**h**) for pure HAP used in toothpaste P2, Rrms = 3.4 ± 1.2 nm.

**Figure 3:**
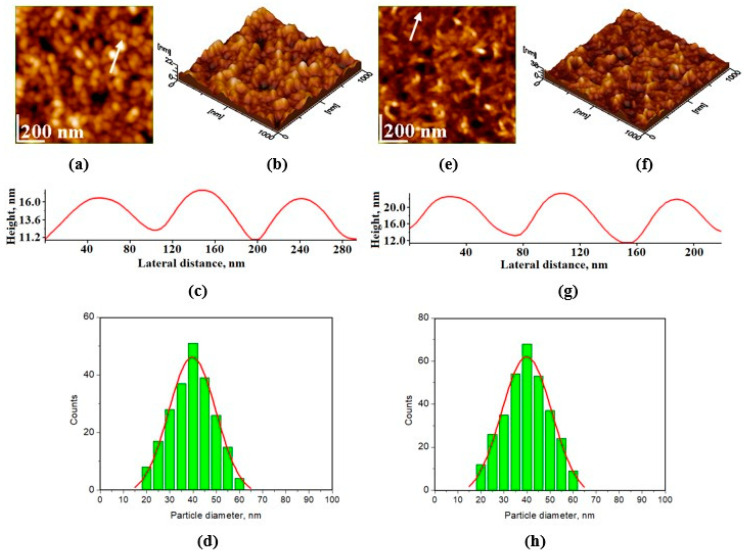
AFM images for HAP-0.23%Mg-3.09%Zn-2%Si-10%Sr used in toothpaste P3: (**a**) a topographic image, (**b**) a tridimensional image, (**c**) a profile along the indicated arrow in panel (**a**), and (**d**) a histogram illustrating the particle size distribution on image (**a**), with a roughness (determined as root mean square), Rrms = 3.7 ± 1.2 nm; the same characteristics: (**e**–**h**) for HAP-2.5%Mg-2.9%Si-1.34%Zn used in toothpaste P4, Rrms = 5.2 ± 2.2 nm. Scanned area 1 µm × 1 µm.

Figure 2 and Figure 3 display AFM images of HAP samples as follows: Figure 2 for HAP-5%Zn used in toothpastes P1 (a–d) and simple HAP used in P2 (e–h); Figure 3 for HAP-0.23%Mg-3.09%Zn-2%Si-10%Sr used in toothpaste P3 (a–d); and HAP-2.50%Mg-2.90%Si-1.34%Zn used in P4 (e–h).

The adsorption of HAP nanoparticles from an aqueous solution onto a solid substrate demonstrates their ability to form self-assembled thin films. This film-forming property is essential when one considers the desired protective layer formed by certain toothpaste formulations on the enamel surface. Here, HAP-5%Zn (P1) demonstrated distinct rounded nanoparticles with diameters of about 40 nm, positioned in a well-structured film (Figure 2a–d), ensuring a uniform surface free of agglomeration clusters. It results in a smooth surface with low roughness, Rrms. Figure 2e–h shows that the simple hydroxyapatite (HAP) used in toothpaste P2 has nanoparticles with rounded shapes and smaller diameters, about 30 nm, that are very uniformly adsorbed onto the glass slide in a smooth and compact thin film, without generating agglomeration clusters.

Figure 3a–d exhibits nanoparticles with rounded shapes and diameters of about 40 nm for HAP-0.23%Mg-3.09%Zn-2%Si-10%Sr, used in toothpaste P3. Their ability to be adsorbed onto a solid substrate is materialized in a very compact, uniform film. This fact influences thin-film roughness, which is slightly higher than in the P2 sample but still lower when compared to P1. Figure 3e–h shows a relatively irregular adsorbed thin film of HAP-2.5%Mg-2.9%Si-1.34%Zn used in toothpaste P4. This is due to the presence of some larger nanoparticles of about 45 nm that are near central points of a uniform network of hydroxyapatite nanoparticles of about 40 nm, which fill the surroundings. This is the reason for which the thin film slightly increases the roughness.

### 3.3. Viscosity of Several Experimental Toothpastes

The four experimental toothpastes used in this study consisted of the following ingredients: double-distilled water (67 wt.%), silicon dioxide (9 wt.%), sorbitol (7.35 wt.%), PEG400 (7.35 wt.%), xanthan gum (0.4 wt.%), birch extract (1.3 wt.%), ethyl alcohol (3.7 wt.%), and sodium lauryl sulfate (0.2 wt.%). The only variation was the active component, hydroxyapatite (4 wt.%), which was employed in the formulations HAP-5%Zn (P1), HAP (P2), HAP-0.23%Mg-3.09%Zn-2%Si-10%Sr (P3), and HAP-2.5%Mg-2.9%Si-1.34%Zn (P4). Viscosity was measured for all toothpastes with two different spindles (#4, #7) and at two spindle speeds, namely 10 and 20 RPM. For reference, two commercial toothpastes, C1 and C2, were also measured in the same conditions. The C2 commercial variety also contained hydroxyapatite. All six tested toothpastes registered values between 80,000 and 100,000 cps.

### 3.4. Nanoscale Remineralization of Human Dental Enamel

**Figure 4:**
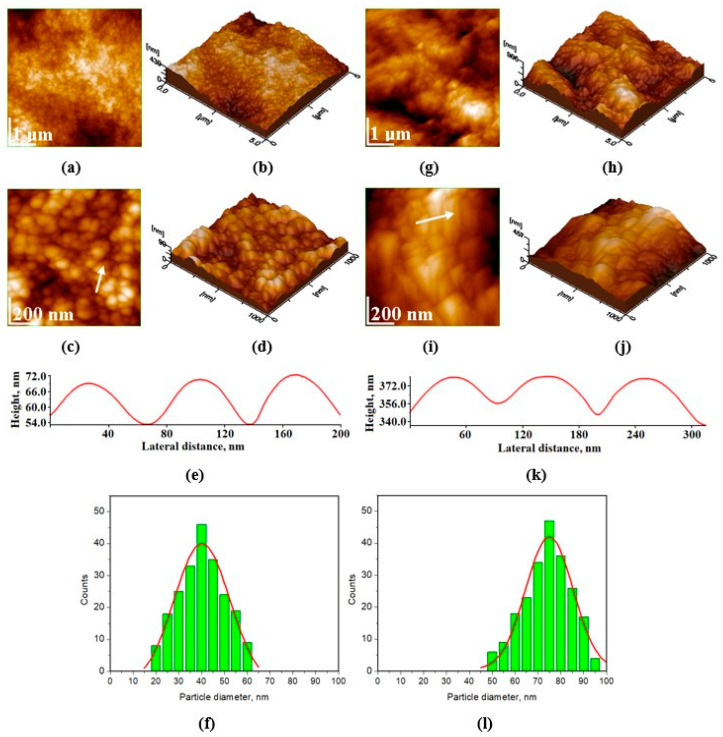
AFM images of healthy enamel, control group (Ctrl): (**a**–**d**), and the artificially demineralized enamel (ADE): (**g**–**j**), where a and g are 2D topographical images on a 5 μm × 5 μm scanned area; c and i are 2D topographical images at 1 μm × 1 μm scanned area; (**b**,**d**,**h**,**j**) are 3D images corresponding to each 2D image; (**e**,**k**) are the profiles along the white arrows in panels c and i, respectively; (**f**,**l**) are the histograms of particle size distribution for each sample (enamel slice), control group (Ctrl) and ADE group, respectively.

Figure 4a–e shows the fine microstructure of the healthy enamel (Ctrl, control group), which is characterized by an excellent cohesion of the mineral material inside as well as between the prisms. The nanoparticles are welded together very well, leading to smooth topography of the enamel slice. Conversely, distinct changes in morphology can be observed in the orthophosphoric acid-etched enamel slice. Following demineralization, the artificially demineralized enamel (ADE) shows a profound disorganization in its surface, which can be observed by strongly individualizing the adjacent areas between two consecutive prisms and by eroding the interior of the prisms (Figure 4g–k). This comes with increased surface roughness values, from the healthy enamel Rrms = 18 ± 2 nm to Rrms = 60 ± 7 nm for ADE, as shown later on in Figure 8B. The histograms in Figure 4f (Ctrl) and 4l (ADE) show a significant increase in HAP size from an average of about 40 nm for normal healthy enamel, Ctrl, to around 75 nm for etched enamel, ADE. 

**Figure 5:**
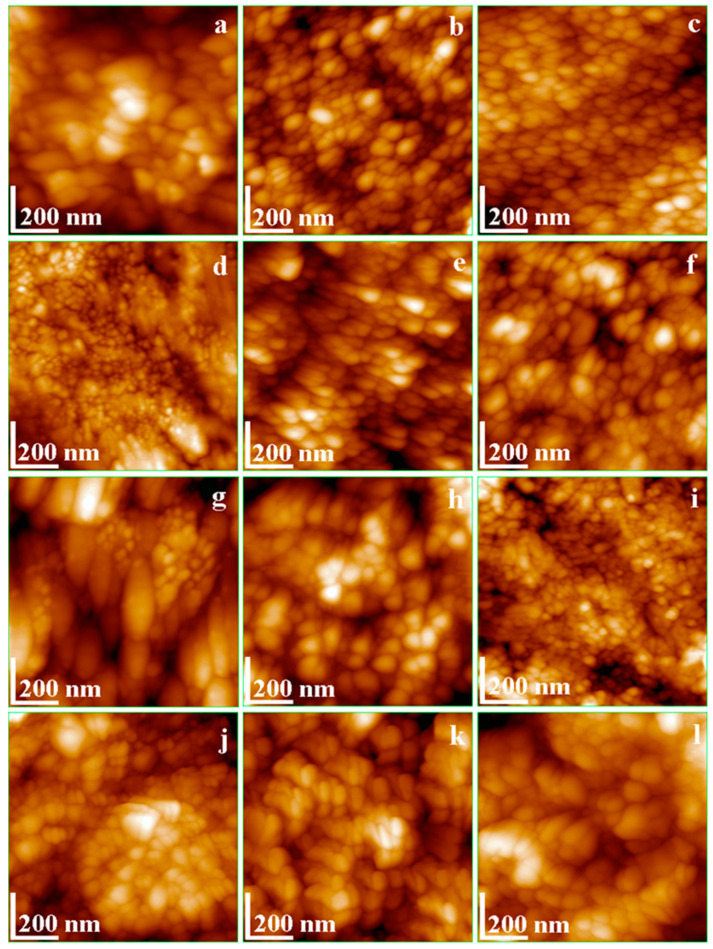
AFM for the 2D topographical nanostructure images showing the nanostructure of the treated ADE slices with toothpastes: (**a**) P1–10 days; (**b**) P1–20 days; (**c**) P1–30 days; (**d**) P2–10 days; (**e**) P2–20 days; (**f**) P2–30 days: (**g**) P3–10 days; (**h**) P3–20 days; (**i**) P3–30 days; (**j**) P4–10 days; (**k**) P4–20 days; (**l**) P4–30 days. Scanned area of 1 µm × 1 µm.

**Figure 6:**
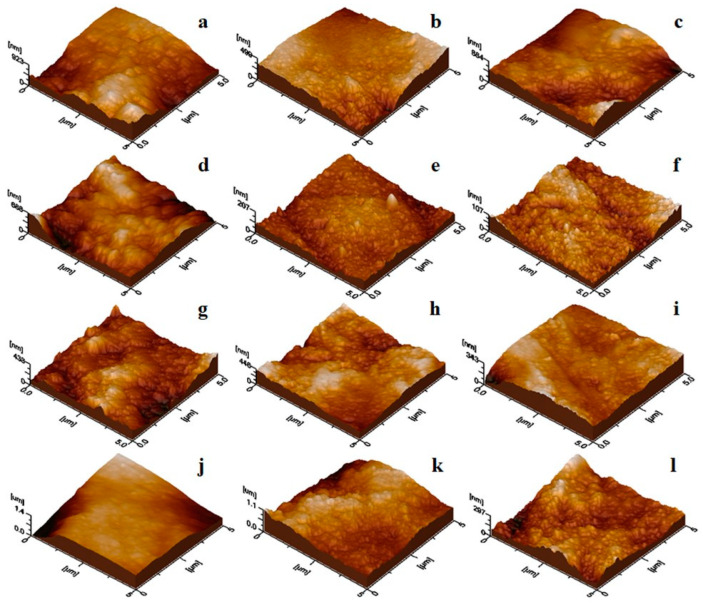
AFM tridimensional images showing the ADE-treated slice nanostructure: (**a**) P1–10 days; (**b**) P1–20 days; (**c**) P1–30 days; (**d**) P2–10 days; (**e**) P2–20 days; (**f**) P2–30 days: (**g**) P3–10 days; (**h**) P3–20 days; (**i**) P3–30 days; (**j**) P4–10 days; (**k**) P4–20 days; (**l**) P4–30 days. Scanned area of 1 µm × 1 µm.

For the observation of the remineralization progression, Figure 5 offers the topographic images for treated samples (scanned area 1 µm × 1 µm). The enamel coverage with newly added HAP nanoparticles, as well as its uniformity and compactness, are more easily observable at this larger magnitude, which is necessary to establish a hierarchy among the used toothpastes. Both the AFM topographical images in Figure 5 and their three-dimensional counterparts in Figure 6 show the evolution of the nanostructure morphology of the samples during the treatment stages. The shape and size of the HAP nanoparticles that remineralized the ADE surface, the uniformity with which they deposited on the surface, and the compactness of the deposited remineralization layer are the morphostructural factors that characterize the success of the remineralization treatment (in other words, the density of nanoparticles in the adsorbed HAP layer on the ADE surface).

Even after only 10 days of therapy, all toothpastes are quite effective in remineralizing the ADE samples. However, by continuing the treatment up to 20 days, better uniformity in the scanned enamel (ADE slices) samples was observed. Further, built on ADE slices was an excellent self-assembled layer that was even more consolidated after an additional 10 days of treatment (at about 30 days) for all toothpastes. 

**Figure 7:**
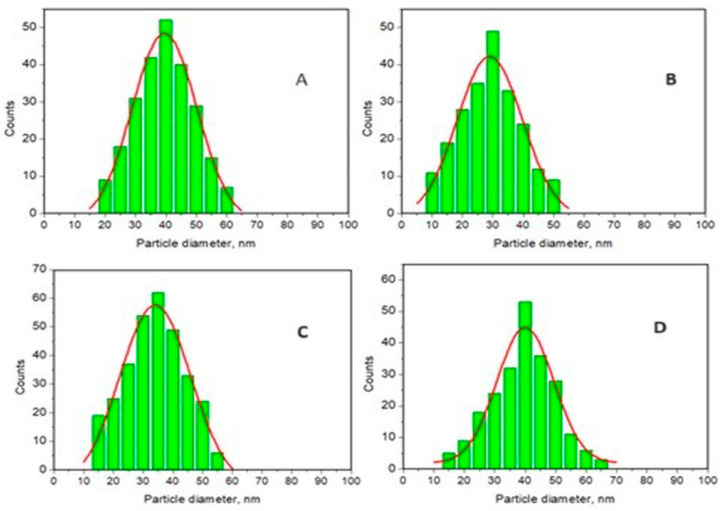
Particle distribution histograms and Gauss curves for selected AFM images of treated ADE slices with toothpastes at 30 days: (**A**) histogram of the nanostructure for P1 treatment shown in Figure 5c; (**B**) histogram for P2 treatment shown in Figure 5f; (**C**) histogram for P3 treatment shown in Figure 5i; and (**D**) histogram for P4 treatment shown in Figure 5l.

The histograms corresponding to the ADE-remineralized samples after 30 days of treatment, indicated in Figure 5c,f,i,l, are shown in Figure 7.

**Figure 8:**
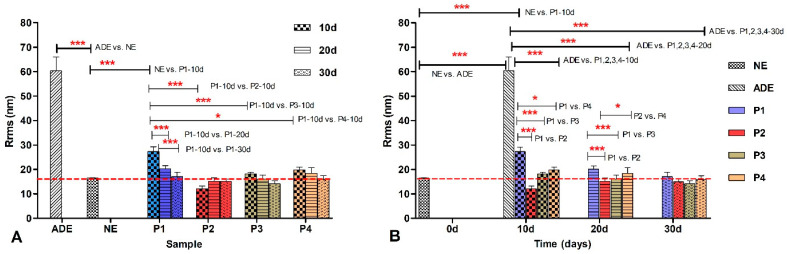
(**A**) Graphical representation of the Rrms values obtained by AFM (on 1 µm × 1 µm scanned area) as a function of the enamel samples for artificially demineralized enamel, ADE, NE, and ADE samples at 10, 20, and 30 days of treatment with tested toothpastes P1, P2, P3, and P4. (**B**) Comparison of Rrms values for enamel samples ADE, NE, and ADE treated with toothpastes P1, P2, P3, and P4, and subsequently noted P1, P2, P3 and P4, versus time, at 10, 20, and 30 days of treatment. The following degrees of statistical significance are denoted by stars: * 0.01 < *p* < 0.05; *** *p* < 0.001.

To compare the roughness, Rrms in nm, data of the remineralization process dynamics induced by treatments of enamel samples using all toothpastes, statistical analysis with one–way ANOVA was applied with an assessment of differences among artificially demineralized enamel (ADE), natural (healthy) enamel (NE), and among P1–P4 treated enamel samples. The statistics are presented in Figure 8A,B. 

In Figure 8B, where the Rrms data were grouped according to time, the best remineralization level was achieved by P2 almost equally with P3 and followed by P4, and P1. There are observed statistical differences between P1 vs. P2 at 10 and 20 days (p***) and between P1 vs. P3 at 10 and 20 days (p***). At 30 days all toothpastes were reaching the values of the control (healthy) enamel.

## 4. Discussions

Several studies investigated the remineralization capability of commercial toothpastes using various analytical techniques. A comprehensive array of analytical techniques, including spectroscopic methods such as ATR-FTIR and Raman microscopy, as well as imaging techniques like HV-SEM/EDX [75] environmental scanning electron microscopy (ESEM) [76,77] and atomic force microscopy (AFM) [71,72] were employed to provide detailed characterization of dental samples, offering insights into both their chemical composition and structural morphology. Although complementary information is given by these techniques, there are differences as to the conditions under which they can be applied and the data that can be obtained.

In this study, XRD was used [69,70] to characterize the hydroxyapatites synthesized in our laboratory that are the basis of toothpaste. AFM was applied to highlight the mineralization pattern.

XRD patterns, provided in Figure 1, confirmed the presence of a unique phase of pure stoichiometric HAP structure for all synthesized msHAP. These nanoHAPs may inherit the characteristics of hydroxyapatite and can be impacted by the substituting functional elements (e.g., Mg, Zn, Sr, and Si).

The evaluation of crystallite size employed the Scherrer relation: D = (kλ/βcosθ), where k = 0.9, λ = 1.54056Å, β represents the full width at half maximum (FWHM) corrected for instrumental broadening, and θ denotes the diffraction angle for the selected diffraction line (0 0 2) positioned at 2θ = 25.900°. The resultant crystallite size, lattice parameters (a, b, c), and crystallinity are presented in Table 1. Pure hydroxyapatite (HAP) exhibits the largest crystallite sizes, 414 Å, whereas the smallest sizes are observed for HAP substituted with Mg-Zn-Sr-Si (325 Å). Although lattice parameters exhibit minimal variance across samples, HAP-Mg-Zn-Sr-Si attains the highest values due to the larger ionic radius of Sr ions in comparison to Ca ions. 

Following the size distribution of HAP nanoparticles and their behavior as seen through AFM imaging, one could predict that simple HAP would have the best behavior when incorporated into toothpaste, as the particles are smaller and much more uniformly arranged. This would fall in line with the most important step in generating proper enamel remineralization, namely, uniform nanoparticle adsorption. The adsorbed nanoparticles must be bonded to the enamel structure via local epitaxial regeneration, which ensures that the hydroxyapatite crystal lattice within the nanoparticles is bonded to the enamel crystal lattice. In contrast, larger nanoparticles tend to fill deeper gaps and smaller nanoparticles surround them when adsorbed onto the surface of glass, ensuring optimal smoothness of the deposited HAP layer. This characteristic may also be advantageous when performing adsorption on uneven surfaces such as demineralized enamel. This disposition for all samples could be better observed in 3D imaging, for instance, as shown in Figure 2b,f and Figure 3b,f.

Viscosity is an important parameter concerning toothpastes, as it has an impact on spreadability, thickness, tube extrusion, and shape retention [67,68]. A general tendency that can be observed for all toothpaste samples is a decrease in viscosity values with the increase in spindle speed. This observation is in accordance with data reported in the literature [78,79]. A desired viscosity range for toothpaste is typically 60,000–240,000 cps [80], although values do tend to vary across the scientific literature in accordance with proposed compositions. All the analyzed toothpastes have similar values of viscosity and fit in with comparable data quoted in the literature. Since all proposed toothpastes have identical basic compositions (wt.%), the variation in values can only be attributed to the type of hydroxyapatite used and the influence of specific substitution ions. Sample P2, which contains unsubstituted hydroxyapatite (HAP), led to the lowest values in viscosity, followed by sample P1, which contains monosubstituted hydroxyapatite (HAP-5%Zn), then P3 (HAP-0.23%Mg-3.09%Zn-2%Si-10%Sr), and lastly P4 (HAP-2.5%Mg-2.9%Si-1.34%). Thus, it is conceivable to say that an increase in substitution ions would lead to an increase in viscosity. 

HAP crystals in most dental enamel are susceptible to acid, affecting their structure. Hydroxyapatite in enamel is stable at pH 7.4, but when pH drops below 5.5, mineral dissolution occurs, leading to enamel erosion [79,80]. Acidic beverages exacerbate this erosion, creating an environment for demineralization, resulting in a honeycomb structure formation due to mineral loss [81,82]. During acid etching (e.g., orthophosphoric acid), both organic and inorganic enamel components undergo dissolution, with water-promoting acid diffusion into and mineral content diffusion out from enamel. This dissolution occurs preferentially in central defects on crystal axes, creating specific topography in etched enamel, with varying depths depending on crystal orientation [83].

Remineralization of enamel involves restoring mineral ions to the hydroxyapatite structure, potentially reversing early-stage dental caries. This process occurs naturally under near-neutral pH conditions when calcium and phosphate ions from saliva and plaque fluid redeposit within the caries lesion, leading to the formation of larger, more acid-resistant hydroxyapatite crystals [84,85,86]. 

As observed in Figure 4 for the demineralized enamel, the measured nanoparticles appear larger than those of healthy enamel. The partial dissolution of the protein binder of hydroxyapatite crystallites, which tends to gain some space between them, causes the nanostructured HAP unit diameter to increase because of demineralization [87]. The space between enamel prisms is typically occupied by water and organic components. However, there is another aspect to consider when using orthophosphoric acid, namely that hydroxyapatite reacts with it, leading to a compound, Ca(H_2_PO_4_)_2_, which is soluble in water (Equation (1)).

Ca_10_(PO_4_)_6_(OH)_2_ +14H_3_PO_4_ => 10Ca(H_2_PO_4_)_2_ +2 H_2_O (1)

After 10 days of treatment, all toothpaste samples improved the microstructure of demineralized enamel (Figure 5a,d,g,j), likely due to hydroxyapatite’s self-affinity, interacting with biological HAP in enamel. Toothpastes with three and four ionic substitutions in the HAP lattice showed good but not excellent results. However, toothpaste P1, containing HAP-5%Zn, produced poorer results due to partial coverage of demineralized areas and poor attachment of the new HAP nanoparticles. Further treatment is hypothesized to be necessary. Notably, toothpaste P2, containing simple hydroxyapatite, showed the best coverage of demineralized areas, with high cohesion and surface uniformity. Continuing treatment for another 10 days resulted in further improvement, resembling healthy enamel morphology (Figure 5b,e,h,k). While successful remineralization was achieved after 20 days, extending treatment to 30 days allowed for observation of toothpaste behavior over an extended period. This may lead to consolidation of deposits formed in the first 20 days, with the deposit layer becoming thicker and more resistant to enamel demineralization. This progression in remineralization is illustrated in the 3D topographical images provided in Figure 6.

It is important to point out that the average diameter of HAP nanoparticles obtained from histograms given in Figure 7 are in the series of HAP (33.0 ± 3) < HAP-Mg-Zn-Sr-Si (38.9 ± 5) = HAP-Mg-Zn-Si (39.0 ± 4 < HAP-Zn (41.6 ± 4) or P2 < P3 = P4 < P1, which is similar to toothpaste efficacy, justifying the role of nanoparticle size of HAPs in the remineralization process dynamics. 

Untreated healthy enamel typically has HAP nanoparticles with a diameter of approximately 40 nm. After just 10 days of treatment, all toothpastes show promising restorative effects, with enamel surface values significantly lower than artificially demineralized enamel (Figure 4). Toothpaste P1, containing HAP-5%Zn, exhibits the most notable improvement, with particles decreasing from around 45 nm at 10 days to approximately 40 nm at 20 days, consolidating at 30 days. However, surface coverage is equally crucial. Toothpaste P2, with simple hydroxyapatite, maintains stable 30 nm nanoparticles over 10 to 30 days, accompanied by the best coverage among the samples. Toothpastes P3 and P4, containing HAP substituted with four and three different ions, respectively, exhibit similar nanoparticle size values to those of the original HAP powders, indicating a successful attachment of these HAP nanoparticles to the enamel surface during remineralization treatment.

Furthermore, our results showed that there were differences in artificially demineralized enamel morphology after toothpaste treatments, requiring the rejection of the study’s null hypothesis. Also, it was demonstrated that each toothpaste formulation was able to induce a new layer deposited on the treated enamel surface, but currently, the chemical composition of the formed layers is still under investigation using various methods, including SEM and EDX. 

While the toothpaste compositions are very efficacious for remineralization purposes and the results are quite promising, a possible future study should include demineralization–remineralization cycles, to better mimic in vivo conditions. Different types of acidic environments should also be included, with the incorporation of acidic beverages that are highly consumed among the population. 

Another future research direction would be to translate laboratory findings into in vivo and clinical research to validate the efficiency of diverse toothpaste formulations for enamel remineralization under realistic conditions using controlled trials and long-term toothpaste performance assessments. Also, it should be investigated whether specific brushing techniques or application frequency for toothpaste formulations containing hydroxyapatite nanoparticles can improve the remineralization process.

## 5. Conclusions

In this study, we formulated four toothpastes with high enamel remineralization potential, containing both hydroxyapatite (HAP) and substituted HAPs, of very high purity, obtained by mild calcination at 300 °C for 1 h, to completely remove the impurities. Results indicated excellent enamel restoration with all formulations. After just 10 days of treatment, artificially demineralized enamel showed nanostructure normalization, nearing values of healthy enamel. Extended treatment improved enamel surface coverage, followed by consolidation of HAPs nanoparticles within the (self-assembled) layer deposited on the enamel surface. Toothpaste P2, with simple HAP (average size of about 30 nm), yielded the best results, closely followed by P3 (average size of around 40 nm) and P4 (average size of about 40 nm). Although toothpaste P1 (HAP-5%Zn) also showed outstanding results, it required additional treatment time. After 30 days, all toothpastes completely remineralized the enamel, which is a crucial finding for future studies. The presence of HAP nanoparticles in each toothpaste facilitated uniform enamel remineralization through adsorption of these HAP nanoparticles onto the enamel surface. Morphological features such as the shape and size of HAP nanoparticles played a significant role in the enamel repair process. Birch extract in the toothpastes contributed to infection prevention and caries protection. Overall, the AFM technique proved to be effective in investigating enamel remineralization by its capability in assessing morphological changes and roughness of the nanosurface.

## Figures and Tables

**Table 1:** Crystallite size, crystallinity degree, and lattice parameters for the calcined hydroxyapatites, HAPs, used in toothpastes P1–P4.

Hydroxyapatites, HAPs	HAP	HAP-Zn	HAP-Mg-Zn-Si	HAP-Mg-Zn-Sr-Si	
Toothpastes	P2	P1	P4	P3	
Crystallite size (Å)	414	332	328	325	
Crystallinity (%)	38.9	32.7	31.8	30.5	
Lattice parameters:					
a = b (Å)	9.431	9.422	9.432	9.434	
c (Å)	6.877	6.861	6.881	6.885	

## Data Availability

Data are available on request.
